# The antagonistic dance between two ER-shaping proteins in plant cells

**DOI:** 10.1093/plphys/kiad593

**Published:** 2023-11-07

**Authors:** Yang Shao, Jiaqi Sun

**Affiliations:** The Key Laboratory of Plant Development and Environmental Adaptation Biology, Ministry of Education, School of Life Sciences, Shandong University, Qingdao 226237, China; Assistant Features Editor, Plant Physiology, American Society of Plant Biologists; The Key Laboratory of Plant Development and Environmental Adaptation Biology, Ministry of Education, School of Life Sciences, Shandong University, Qingdao 226237, China

The endoplasmic reticulum (ER) serves as a critical hub for lipid and protein biosynthesis, making it indispensable for plant cellular growth and adaptability to external stresses. The ER structure is composed of flattened sheets and long cylindrical tubules with high membrane curvature. Through continual remodeling, the ER adjusts its structure to meet the fluctuating biosynthetic demands in a process that involves an intricate network of regulatory mechanisms (Brandizzi 2021).

A key mechanism underlying this structural dynamism is ER fusion, resulting in the rapid formation of 3-way tubular junctions that are branch points in the ER network (Chen et al. 2011). In *Arabidopsis*, the ER “fusogen” protein RHD3 has been shown to contribute to ER fusion, and its activity was finely modulated by the ubiquitination mediated by Lunapark proteins (Sun et al. 2020). Studies in other systems have implicated reticulon proteins (RTNs) as another vital set of actors in this process because they induce and maintain the high curvature of ER tubules (Hu et al. 2008). However, the interaction between reticulon proteins and RHD3 homologs is not clear because it can appear to be synergistic or antagonistic. For example, in yeast a homolog of RHD3 works in synergy with Rtn1 to shape the ER (Hu et al. 2009), and in plants it has been reported that RHD3L2 and RTNLB13 physically interact and act cooperatively to determine ER morphology (Lee et al. 2013). On the other hand, in mammals and *Drosophila*, RHD3s homologues have been shown to counterbalance the effects of reticulons in ER connectivity (Wang et al. 2016; Espadas et al. 2019).

Given these varying roles across different organisms, the functional significance of the interaction between RHD3 and RTNs remains enigmatic. Specifically, it is unclear whether these proteins work in synergy to shape the ER or counteract each other's functions.

In this issue of *Plant Physiology*, Wang and Zheng (2023) offer insights into the complex relationship between RTNs and the ER membrane fusogen protein RHD3 in plant cells. One of the paper's most significant discoveries is that RTNLB3 serves to inhibit the fusion activity of RHD3, likely by interfering with the dimerization of RHD3, a process integral to its fusion function (Fig). This inhibition was demonstrated through a series of experiments, including quantitative ER fusion assays in yeast, transient overexpression in plant cells, Bimolecular Fluorescence Complementation (BiFC) assays for RHD3 dimerization, and yeast 2-hybrid experiments. The data demonstrate the antagonistic regulatory role of RTNLB3 on RHD3's ER fusion activities. Notably, the authors reveal that the RTNLB3 physically associates with RHD3 and its homolog RHD3L2 at specific locations along the tubular ER as well as at 3-way junctions. This finding uncovers the nuanced spatial regulation of RHD3-mediated ER fusion by RTNs and suggests that this regulation may occur through the prevention of RHD3 accumulation at locations where 3-way junctions—or ER membranes—are poised for fusion (Fig).

**Figure.**
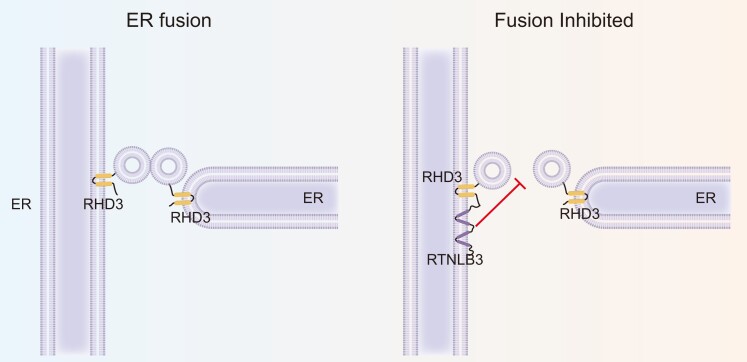
Inhibition of RHD3-mediated membrane fusion by RTNLB3. Initially, RHD3 molecules accumulate at the site targeted for membrane fusion. Subsequently, a conformational change in the RHD3 proteins tethers the membranes together, leading to the formation of a new 3-way junction. However, this process is disrupted by RTNLB3, which inhibits the critical dimerization of RHD3 essential for membrane fusion. The exact mechanism through which RTNLB3 interferes with RHD3's dimerization remains undetermined.

Employing knockout studies, the authors showed that the absence of RTNLB3 results in a predominance of sheet-like ER structures within cells, thereby confirming its crucial role in maintaining a tubular ER morphology. Further substantiating this, subcellular localization studies showed that RTNLB3 is localized to the tubular ER, laying the groundwork for further exploration into the functional interplay between RTNLB3 and RHD3.

Interestingly, the genetic removal of RTNLB3 partially rescues the developmental and ER morphological defects observed in the *rhd3* knockout mutant. This genetic evidence further fortifies the concept of an antagonistic functional relationship between the 2 proteins and underscores the in vivo significance of balancing ER fusion and tubulation activities, as mediated by RHD3 and RTNs.

The authors also delve into the mechanistic aspects of these interactions. They identify the first 2 transmembrane domains of RTNLB3 as essential for its functional interaction with RHD3, providing key insights into the molecular underpinnings of this relationship. This leads them to propose several plausible models that could explain the dynamic regulation of these interactions in shaping the ER.

In summary, this study substantially deepens our understanding of the molecular interplay between 2 primary classes of ER-shaping proteins in plants. The revelation of the balance and spatial regulation between ER fusion and tubulation not only has broad implications for the control of ER morphology but also paves the way for future research into the coordinated activities of RHD3 and reticulons in shaping the ER network.

## Data Availability

No new data were generated or analyzed in this manuscript.

## Dive Curated Terms

The following phenotypic, genotypic, and functional terms are of significance to the work described in this paper:

RHD3 Gramene: AT3G13870
RHD3 Araport: AT3G13870
